# Diabetic ketoacidosis with central nervous system involvement: Conventional and advanced magnetic resonance neuroimaging findings

**DOI:** 10.1177/19714009241248745

**Published:** 2024-04-23

**Authors:** Luca Caschera, Giorgio Fiore, Simone Nava, Stefania Criscuolo, Francesco M Lo Russo, Silvia Casale, Giorgio Conte, Giulia Platania, Antonella Costa, Giorgio Carrabba, Marco Locatelli, Fabio Maria Triulzi

**Affiliations:** 1 Neuroradiology Unit, 9339 Fondazione IRCCS Ca’ Granda Ospedale Maggiore Policlinico, Italy; 2 Unit of Neurosurgery, 9339 Fondazione IRCCS Ca’ Granda Ospedale Maggiore Policlinico, Italy; 3 Unit of Oncology and Hemato-Oncology, University of Milan, Italy; 4 Postgraduational School of Radiodiagnostics, University of Milan, Italy; 5 “Aldo Ravelli” Research Center for Neurotechnology and Experimental Brain Therapeutics, University of Milan, Italy; 6 Department of Pathophysiology and Transplantation, Università Degli Studi Di Milano, Milan, Italy

**Keywords:** Diabetic ketoacidosis, brain, magnetic resonance imaging, functional MRI, diffusion tensor imaging

## Abstract

Diabetic ketoacidosis (DKA) is a serious complication in children with diabetes mellitus type 1 (DM1). In rare and severe cases DKA may be complicated by cerebral edema, central brain herniation and cerebral infarctions. We present the magnetic resonance imaging findings in a child with DKA and central nervous system involvement; diffusion tensor imaging (DTI) and functional MRI (fMRI) were performed to assess the white matter integrity of sensory pathways and cortical sensory processing. Conventional imaging showed bilateral uncal herniation, effacement of the perimesencephalic cisterns, wide ischemic lesions in the posterior cerebral artery (PCA) territories, sagging brainstem and Duret’s hemorrhage consistent with signs of central brain herniation and intracranial hypertension. Advanced MRI showed a possible left-sided cortical reorganization for sensory function, with underlying left cortico-talamic and cortico-spinal pathways less severely impaired. Knowledge of the full framework in these conditions is of vital importance for timely patient management; advanced neuroimaging techniques may be considered as prognostic indicators in those cases with extensive involvement of eloquent brain areas.

## Introduction

DKA is one of the most serious complications of DM1, it is a complex metabolic state characterized by dehydration and electrolyte disturbances, hyperglycemia, anion gap metabolic acidosis and ketonemia leading to coma and severe cerebral edema.^1,2^ Central brain herniation with subsequent development of ischemic or hemorrhagic stroke is a rare complication that results in a significant mortality and high long-term neurological sequelae.^3^ We present conventional and advanced neuroimaging findings in a pediatric patient with DKA.

### Case report

A 9 year-old boy with persistent poorly controlled DM1 was admitted to our pediatric neuro-intensive care unit with DKA and a Glasgow Coma Scale (GCS) of 6. During hospitalization, the child presented with spastic quadriplegia in decortication and retraction of the four limbs to painful stimulation. Baclofen was introduced for spasticity. Because of autonomic crisis, Midazolam and Diazepam were administered at first, and then gradually suspended with the introduction of Clobazam. A tracheostomy was performed after 7 days, and the patient remained eupneic. We faced a difficult glycemic control, with the need for extemporaneous insulin boluses to maintain blood sugar levels inferior to 300 mg/dl. The switch to enteral nutrition via percutaneous endoscopic gastrostomy (PEG), in association with subcutaneous insulin administration, was effective for glycemic control. A ventricular-peritoneal shunt was placed for elevated intracranial pressure (ICP) values (23 mmHg on continuous recording lasting 1 h).^4^ Brain computed tomography (CT) and brain magnetic resonance imaging (MRI) were performed at admission and during hospitalization. In addition to conventional imaging, advanced imaging sequences were acquired to better characterize the neural substrates of potentially residual sensorimotor function.

#### CT and conventional MR imaging findings

**Figure 1.**
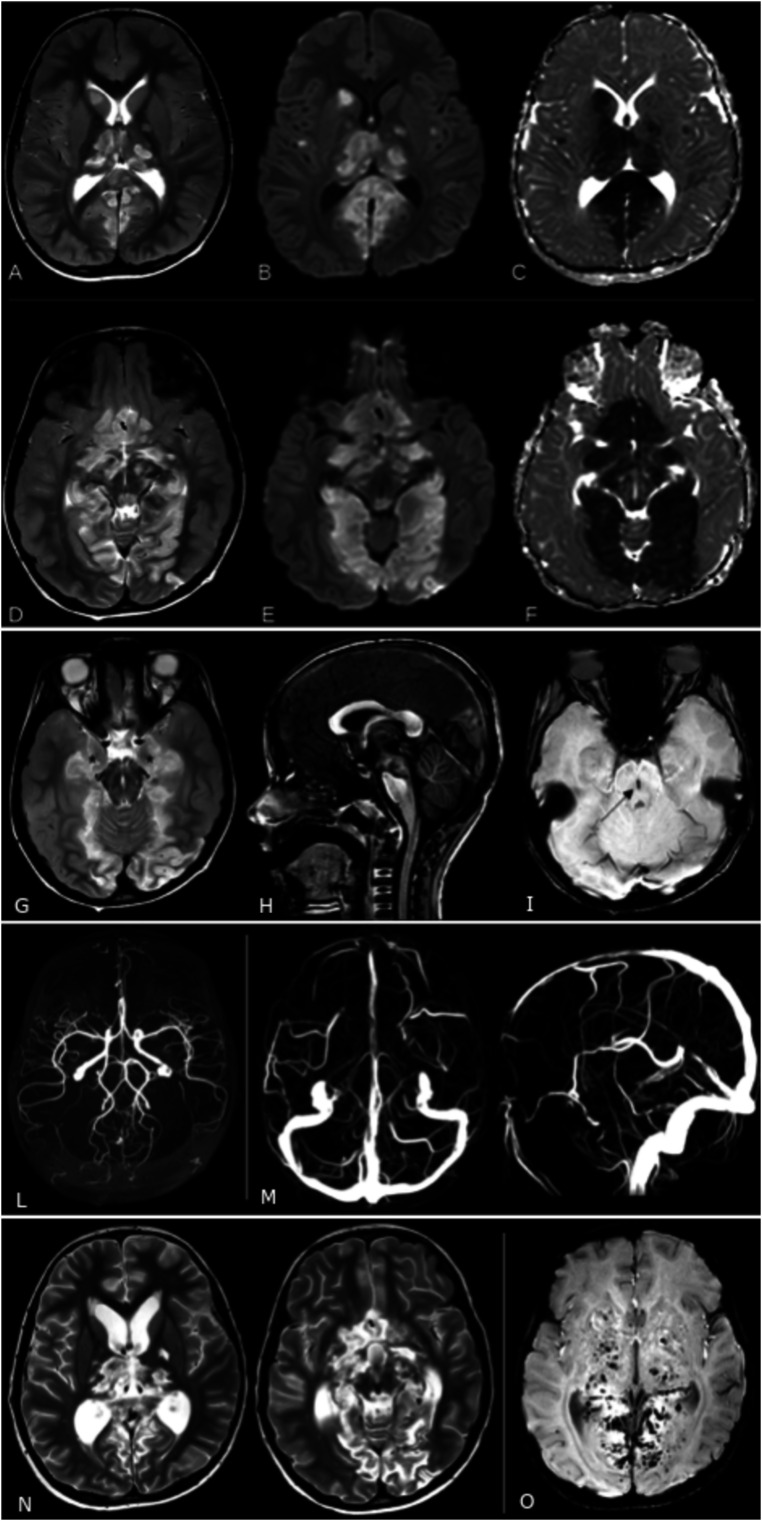
Axial (a, d) TSE T2, (b, e) DWI and (c, f) ADC sequences showing the presence of large areas of T2 hyperintensity and restricted diffusion consistent with striking parenchymal ischemia. (g) Axial TSE T2 sequence showing mild bilateral uncal herniation (asterisks) and effacement of the perimesencephalic cisterns. (h) Sagittal 3D TFE T1 sequence showing a sagging brainstem with midbrain descent through the tentorial incisura. (i) Axial SWI sequence showing a midbrain sagittal hypointense stripe (arrow) consistent with the presence of hemosiderin components (Duret’s hemorrhage). (l) 3D time of flight angiography and (m) phase contrast venography showing no abnormalities with the regular representation and patency of the main arterial and venous intracranial vessels. Bottom row: follow-up MRI performed 30 days after admission. (n) Axial TSE T2 sequence showing the malacic evolution of the parenchymal lesions and the dilatation of the ventricular system. (o) Axial SWI sequence showing the presence of multiple microhemorrhages.

The brain CT scan showed signs of intracranial hypertension with diffuse sulcal effacement, reduced visualization of the basal cisterns, a slit-like third ventricle, the presence of subtle hypodensity and swelling of the thalami and occipital lobes due to ischemia in the PCA territories secondary to previous central brain herniation and arterial compromise. Brain MRI showed the presence of diffuse cerebral edema. There were also large confluent areas of T2-FLAIR hyperintensity and restricted diffusion in the thalami, hypothalamus, optic chiasm, anterior perforated substance, fornix, sub frontal area, cerebral peduncles, and periaqueductal gray matter, globi pallidi and caudate nuclei, hippocampi/parahippocampal gyri and posterior cingulate gyri, occipital lobes and splenium of the corpus callosum (Figure 1(a-f)). Furthermore, mild bilateral uncal herniation effacing the perimesencephalic cisterns, sagging brainstem, and midbrain descent through the incisura with signs of Duret’s hemorrhage were found (Figure 1(g-i)). Three-dimensional time of flight magnetic resonance angiography (3D-TOF MRA) and phase contrast magnetic resonance venography (PC-MRV) showed no abnormalities (Figure 1(l-m)). Follow-up brain MRIs showed the malacic evolution of the parenchymal lesions and a progressive dilation of the supratentorial ventricular system (Figure 1(n-o)).

#### Advanced MR imaging: Techniques and findings (DTI and fMRI)

##### Materials—Advanced MRI (DTI and fMRI)

The integrity of white matter sensory pathways was studied through diffusion tensor (DTI), while cortical sensory processing was assessed by functional MRI (fMRI). MR imaging was performed using a 3 T scanner (Achieva, Philips Healthcare, Best, the Netherlands). The exam included a DTI sequence with 64 diffusion encoded directions and three fMRI sequences with tactile stimulation of the patient’s hands and feet in a block design alternating 15s on/off stimulation. The DTI processing pipeline was performed using Tortoise (Pierpaoli et al. 2010), Diffusion Toolkit, and Trackvis (Wang et al. 2007) software: realignment, registration to the anatomic T1 volume, motion and eddy current distortions correction, as well as an upsampling of diffusion images to a final voxel size of 1.5 mm, were computed. Virtual dissection of bilateral corticospinal tract (CST), as well as arcuate fasciculus (AF) in its three segments (frontoparietal, frontotemporal and temporoparietal as described in Catani, 2005) were performed. fMRI data were imported in Brain voyager software (Brain Innovation, Maastricht, the Netherlands) and underwent preprocessing steps including 3D motion correction and spatial smoothing. Activation maps for the left and right sides for each stimulation area were produced.

##### Results—Advanced MRI (DTI and fMRI)

**Figure 2.**
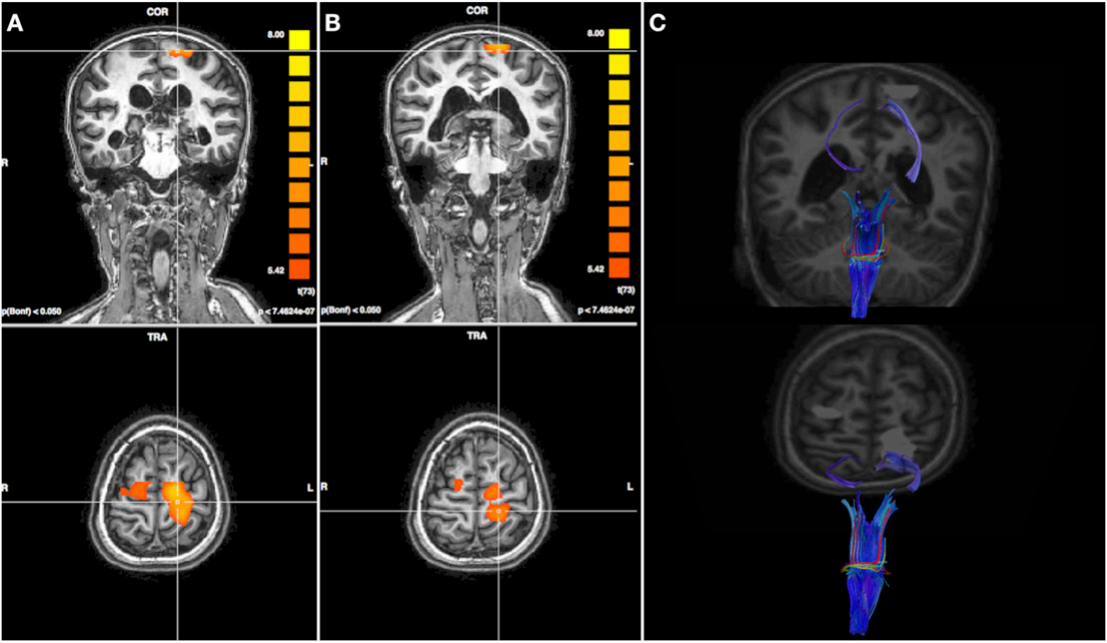
(a) fMRI activation maps for right foot tactile stimulation on T1 axial and coronal views. (b) fMRI activation maps for left foot tactile stimulation in the same projections. (c) tractographic reconstruction showed partial impairment of sensory white matter pathways, with a higher extent in the right one, consistently with more severe involvement of the homolateral thalamus as revealed by conventional MRI. Functional activation clusters are superimposed as well (in light grey).

The DTI analysis revealed that the described thalamic lesion affected the thalamic-cortical projections of somatosensory white matter pathways: in their tractographic representation they appeared interrupted, whereas the spinal-thalamic part appeared intact. A slight left/right difference has been observed, with thinner right cortical projections compared to left, consistently with the conventional MRI showing right thalamus more severely lesioned. On the left side, also a higher directional coherence between higher and lower projections was displayed, which could suggest an underlying non-visible residual white matter integrity to support the cortical activity. Regarding fMRI, we considered only the sensory activations derived from feet stimulation, as a significant involuntary head motion during hand stimulation was detected. Tactile stimulation of the right foot produced a robust activation in the left sensorimotor cortex. Interestingly, also the stimulation of the left foot produced an overlapping activation in the same left, ipsilateral region. Although to a smaller extent, the ipsilateral cortical activation for the left foot was obtained at the same statistical threshold (<0.001) and after Bonferroni correction for multiple tests. These results suggested selective damage to the right subcortical sensory pathways and a possible contralateral cortical reorganization for tactile information processing (Figure 2).

### Discussion

DKA is a well-known complication of DM1 that occurs in approximately one-third of DM1 children younger than 5 years.^5^ In rare cases, it may cause severe injury to the CNS with poor long-term neurocognitive and sensorimotor outcomes. Cerebral edema, central brain herniation and diffuse ischemic-hemorrhagic damage are well-known feared consequences.^2,7^ According to the literature, the possible pathomechanisms of brain injury could be secondary to a combination of intracranial hypertension and metabolically induced proinflammatory state with vascular endothelial dysfunction. The metabolic imbalance is responsible for hyperosmolarity and increased blood viscosity, vascular endothelial damage, acidemia-related stiffness of red blood cells and hyperglycemic-induced vasoconstriction, brain edema with subsequent intracranial hypertension causing mechanical compression/stretching of the major vessels.^6–8^ A systematic conventional imaging description of the possible brain manifestation of DKA is sparse in the literature.^3,9^ Barrot reported two cases of DKA with acute CNS involvement; the main neuroimaging finding was brain edema causing secondary sign of intracranial hypertension including effacement of the sulci and basilar cisternal spaces, decreased size of the cerebral ventricles, ischemic changes in the PCA territories.^8^ In the case reported here, the increased intracranial pressure due to cerebral edema led to uncal herniation, midbrain sagging and severe central brain herniation with subsequent development of large ischemic areas. To better characterize the neural substrates of potentially residual sensorimotor function, advanced imaging techniques were performed. The integrity of white matter somatosensory pathways was studied using DTI and tractography reconstruction, while the cortical sensory processing was assessed by fMRI. Interestingly the tactile stimulation of both feet produced a robust activation in the same left sensorimotor cortex. We surmised that the prevalent damage of the right subcortical sensory pathways, consistent with the conventional MRI finding of the right thalamus more severely affected than the left, led to a possible early cortical reorganization in the left hemisphere, where also the subcortical projection fibers appeared to be more preserved. Figure 2 shows the spatial congruity of the left corticothalamic tract and the cortical activations on the left sensorimotor cortex, elicited by stimulation on both left and right lower limb: this imaging representation might describe the anatomical substrate underlying the function’s plastic reorganization. The findings provided by the aforementioned advanced MRI techniques, documenting the presence of residual somatosensory function with signs of initialized neural plasticity, helped us to better refer the patient to a third-level rehabilitation center.

## Conclusions

DKA is a potentially life-threatening complication of DM1 in the pediatric population; therefore, appropriate knowledge of its MRI findings in brain complications is vital. Advanced neuroimaging techniques can be used as a prognostic indicator and to better characterize potentially residual brain function.
